# Methods of Morbid Histology and Clinical Pathology

**Published:** 1905-09

**Authors:** 


**Methods of Morbid Histology and Clinical Pathology.** By J. Walker Hall, M.D., and G. Herscheimer, M.D. Pp. 290. Edinburgh and London: Wm. Green & Sons. 1905.

This book is well adapted to its purpose, which is to serve as a practical guide for workers in pathological histology and clinical microscopy. The various methods of hardening tissues and of cutting and staining sections for microscopic investigation have now become so multifarious, and in many cases so complicated, that no workers are thoroughly conversant with all of them, nor is it necessary for them to be so. The use of a book of this kind is an absolute necessity when it is desired to employ some unfamiliar process, and it is therefore a most important point that the descriptions of methods should be full and clear, in order that the reader may be able to readily apply them and obtain constant results. We think that the conditions are fulfilled in this work, which gives a very complete and lucid account of the numerous staining processes, not only of those in common use, but of the most recent additions and of the special methods employed for the investigation of special tissues. The procedures for staining micro-organisms and for examining pathological deposits, exudations and secretions, are also included in it, and we can thoroughly recommend the book as a sound practical guide and a most useful addition to the laboratory bookshelf.

